# Lafora Disease Masquerading as Hepatic Dysfunction

**DOI:** 10.7759/cureus.3197

**Published:** 2018-08-24

**Authors:** Faisal Inayat, Waqas Ullah, Hanan T Lodhi, Zarak H Khan, Ghulam Ilyas, Nouman Safdar Ali, Hafez Mohammad A Abdullah

**Affiliations:** 1 Internal Medicine, Allama Iqbal Medical College, Lahore, PAK; 2 Internal Medicine, Abington Jefferson Health, Miami Beach, USA; 3 Infectious Diseases, University of Nebraska Medical Center, Omaha, USA; 4 Internal Medicine, St. Mary Mercy Hospital, Livonia, USA; 5 Pathology, The State University of New York Downstate Medical Center, New York, USA; 6 Internal Medicine, University of South Dakota Sanford School of Medicine, Sioux Falls, USA

**Keywords:** lafora disease, hepatic dysfunction, unusual presentation

## Abstract

Lafora disease is fatal intractable progressive myoclonic epilepsy. It is frequently characterized by epileptic seizures, difficulty walking, muscle spasms, and dementia in late childhood or adolescence. We chronicle here an unusual case of an asymptomatic young male soccer player who presented with elevated liver enzymes. Neurological examination was unremarkable. The diagnostic workup for hepatitis, infectious etiologies, autoimmune disorders, hemochromatosis, Wilson's disease, alpha-1 antitrypsin deficiency, and other related diseases was inconclusive. He subsequently underwent an uneventful percutaneous liver biopsy. Based on the pathognomonic histopathological findings, Lafora disease was considered the likely etiology. The present study is a unique illustration of this rare disorder initially manifesting with abnormal liver enzymes. It underscores the importance of clinical suspicion of Lafora disease in cases with unexplained hepatic dysfunction. Prompt liver biopsy and genetic testing should be performed to antedate the onset of symptoms in these patients.

## Introduction

Lafora disease is a rare, inherited, severe myoclonic epilepsy. In these patients, periodic acid-Schiff (PAS)-positive intracellular inclusion bodies, Lafora bodies (LBs), are the hallmarks of the disease. These can be found in the affected body organs, including the brain, liver, skeletal and cardiac muscles, and sweat glands [1]. The usual onset is at the end of childhood or during early adolescence [2]. The initial clinical features are mostly related to myoclonic epilepsy. The clinical course of the disease is signified by rapid neurodegeneration that mostly leads to death within 10 years after the manifestation of symptoms [3]. Few studies implicated antiepileptic drugs in the treatment of these patients. However, these medications mostly fail to limit the cognitive decline and behavioral changes. Due to these, prompt diagnosis and genetic counseling are particularly warranted. Social support carries paramount importance in the management [4]. Future research should undertake a comprehensive investigation of its pathogenesis as the discovery of a novel treatment for this devastating disease depends entirely on the understanding of its pathophysiology.

The present study involves a unique case of a young male who did not develop any neurological symptoms. He was actually in top physical form, awaiting entry into the army in the Trinidad. It prompts physicians to be vigilant for this serious disease, especially in patients presenting with hepatic dysfunction without an obvious cause. This case was previously presented as an abstract (Abstract: Iqbal S, Ilyas G, Khan Z, Hurairah A, Ferstenberg R. ‘’Soccer Player with Unusual Presentation of Asymptomatic Lafora Body Disease of Liver,’’ Annual Scientific Meeting of the American College of Gastroenterology, October 13-18, 2017 in Orlando, Florida).

## Case presentation

A 22-year-old male was referred to our outpatient hepatology clinic for an evaluation of elevated liver enzymes. The patient was first informed about the hepatic dysfunction one year ago at the time of enlistment in the army in the Trinidad. He had a history of heavy alcohol consumption, at least once every two weeks, for about five years. However, he stopped drinking one year ago. He also reported intermittent, crampy, waxing and waning abdominal pain. Review of the systems was negative for any abnormalities. He was a professional soccer player with excellent health. There was no prior history of drugs, herbal medications, vitamins, or supplements. His mother was diagnosed with breast cancer; however, family history was negative for genetic disorders. On admission, he was hemodynamically stable. Physical and neurological examinations were unremarkable.

Laboratory studies revealed a fasting blood sugar 96 mg/dL (normal, 70-100 mg/dL) and HBA1c 5% (normal, <6%). His complete blood count, hemoglobin, hematocrit, serum albumin, serum electrolytes, renal function tests, and coagulation profile (international normalized ratio, 2.2; prothrombin time, 12 s) were within the normal limits. Serum creatine phosphokinase, aldolase, lactate dehydrogenase, calcium, vitamin D, vitamin B12, and cortisol levels were also within normal ranges. Adrenal and thyroid functions were normal. The details of his liver function testing are provided (Table 1).

**Table 1:** Liver function tests of the patient. Abbreviations and normal ranges: AST, aspartate aminotransferase (normal, <40 U/L); ALT, alanine transaminase (normal, <55 U/L); ALP, alkaline phosphatase (normal, <115 U/L); total bilirubin (normal, 0.0-1.0 mg/dL).

Timing (days)	AST (U/L)	ALT (U/L)	ALP (U/L)	Total bilirubin (mg/dL)
02/09/16	58	80	262	1.5
14/11/16	72	121	251	1
21/11/16	99	147	232	1
21/03/17	59	105	105	1.31

**Figure 1:**
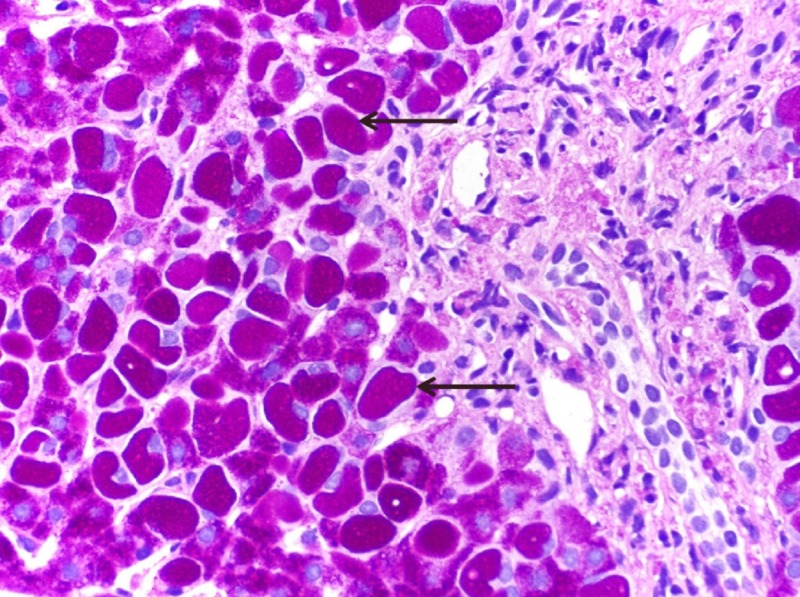
Photomicrograph of histopathological analysis of the liver biopsy specimen showing polyglucosan inclusions (Lafora bodies) in the hepatocytes that are resistant to diastase (Periodic acid–Schiff–diastase staining; 400x). Arrows identify the pathognomic Lafora bodies.

Hepatitis serologies, human immunodeficiency virus, serum ferritin and total iron-binding capacity for hemochromatosis, autoimmune workup, ceruloplasmin for Wilson's disease, and alpha-1 antitrypsin deficiency testing were all negative. Right upper quadrant ultrasound was inconclusive for gross biliary or hepatic abnormalities. Subsequently, an uneventful percutaneous liver biopsy was performed. Pathological examination of the biopsy specimen showed polyglucosan inclusions in the hepatocytes that were resistant to diastase, consistent with LBs (Figure 1).

**Figure 2:**
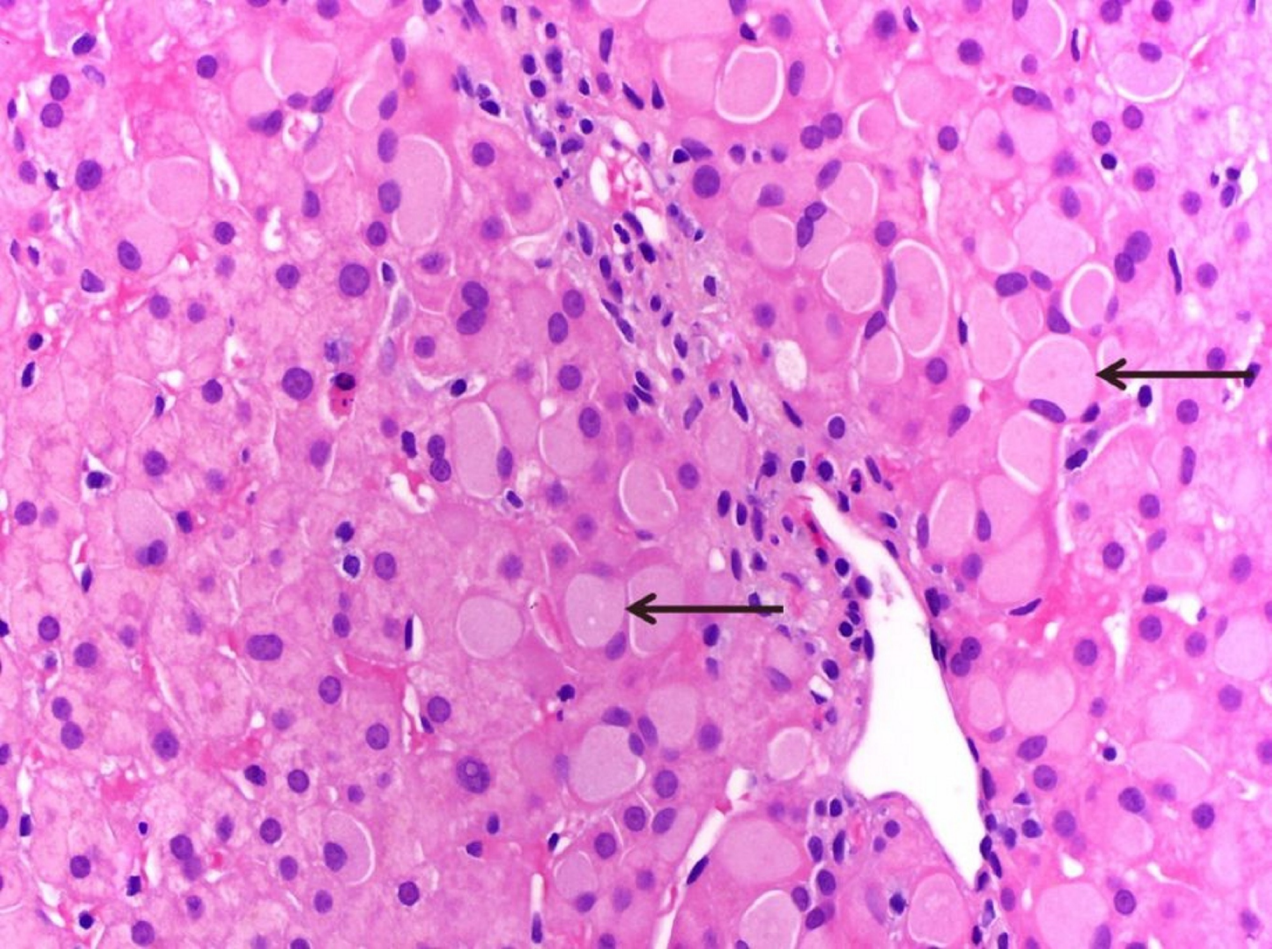
Histpathological examination of the liver biopsy showing periportal hepatocytes with large ground-glass inclusions, consistent with Lafora bodies. (H&E; 400x). Arrows show the pathognomic Lafora bodies.

Periportal hepatocytes had large ground-glass inclusions (Figure 2).

The probable causes were ruled out based on the standard set of investigations. Although it was an atypical presentation, the patient was deemed to suffer from Lafora disease in light of the consistent histopathological features indicating the presence of LBs in the hepatocytes. He was educated about the disease and genetic testing was recommended. However, he had to travel back to the Trinidad and unfortunately, could not come for a follow-up visit.

## Discussion

Lafora disease is an uncommon genetic disorder characterized by progressive myoclonic epilepsy [1]. It was first described in 1911 by Lafora and Glueck in the brain and spinal cord of an adolescent patient who presented with a progressive and fatal form of myoclonic epilepsy [2]. In 1955, Harriman et al. subsequently presented similar inclusions in the heart and liver of one patient with Lafora disease [3]. This clinicopathological entity follows an autosomal recessive pattern of inheritance. Although the exact incidence is unknown, one estimate described the overall frequency of around four cases per million individuals in the world. It more commonly involves the communities with high rates of consanguinity. Therefore, this condition is frequently reported in the Mediterranean countries (Spain, Italy, France), Northern Africa, the Middle East, and in some regions of South India [4].

Lafora disease classically occurs in adolescents from the age of eight years to 19 years (peak: 14-16 years), starting as an action and stimulus-sensitive myoclonus. Focal visual seizures are a typical manifestation presenting as transient blindness or visual hallucinations [4]. Behavioral changes, depression, and apathy are other notable presenting symptoms. Rapid neurological decline ensues within a decade, which is marked by progressing dementia, ataxia, apraxia, cortical blindness, refractory status epilepticus, psychosis, dysarthria, mutism, and respiratory failure that eventually lead to death [4]. Atypical presentation with liver failure has been reported in one patient with his parents remaining asymptomatic. His sibling who was asymptomatic until the age of seven years developed abnormalities in liver function tests and was diagnosed with Lafora disease [5]. Similarly, uncommon presentation with cardiac arrhythmia has also been reported [6]. Two preadolescent asymptomatic patients with abnormalities in liver function tests from one family in which siblings had no symptoms were studied as well; they ended up developing symptomatic Lafora disease in subsequent years [7]. A vast majority of cases have been described in children and teenagers, predominantly with central nervous system manifestations. The present case is unique in this regard as the patient was an adult and sole initial presentation was hepatic dysfunction.

The pathogenesis of this disease remains controversial. It has been explained to be caused by mutations in the *EPM2A* (laforin; a dual specificity protein phosphatase) and *EPM2B* (malin; an E3 Ubiquitin ligase) genes. These genes interact with each other and other proteins to play a critical role in the regulation of glycogen metabolism protecting against accumulations of intracytoplasmic polyglucosan [8]. Contrary to glycogen storage disease type IV, only mild-to-moderate periportal fibrosis has been described in the liver of these patients [9]. The first animal model was studied by deleting the *EPM2A* gene and disease progression was studied by analyzing brain tissue [10]. Furthermore, similar pathology and phenotype were also noted in bench research involving murine models of the disease [4]. The natural progression of Lafora disease has been studied in breeds of dogs revealing the distribution and frequency of LBs in the brain identical to that found in the human disease [11]. However, pathophysiology of this serious disorder warrants meticulous research in future.

The diagnosis can be established by the detection of intra-cytoplasmic basophilic inclusions also called LBs. Lafora disease is clinically similar to Unverricht-Lundborg disease (Baltic myoclonus) but can be distinguished by the advanced age of onset, rapid progression, and history of visual seizures. In the past, the diagnosis was confirmed reliably with the liver biopsy more than the striated muscle biopsy; however, Carpenter and Karpati in 1981 reported diagnostic changes in sweat gland duct cells [12]. In 1987, Busard et al. described the value of careful inspection of the myoepithelial cells of the secretory acini of apocrine glands, especially in the axillary region [13]. Skin biopsy can also detect LBs and it has been considered as a reliable noninvasive approach. On electroencephalography, occipital discharges that arise from a slowed posterior dominant rhythm are highly suggestive of the disease when correlated with the clinical features. Magnetic resonance imaging has limited diagnostic value at the start of the disease. In the current times, pathological diagnosis standard is the presence of LBs in sweat glands. Genetic testing detecting the pathogenic mutation in both the alleles of one of the *EPM2* genes, with the presence of heterozygous mutations in each of the asymptomatic parents, has become the mainstay of the diagnosis [14].

In terms of management, antiepileptic medications such as valproic acid are commonly used to alleviate the burden of the epileptic manifestations [15]. Lamotrigine may help transiently; phenobarbital and primidone are effective, but they have cognitive effects. Levetiracetam has also been empirically employed in adolescents to control the epileptic symptoms. Similarly, topiramate and zonisamide have also shown antimyoclonic activity in some patients [15]. Recently, a study by Goldsmith and Minassian highlighted the use of perampanel, which is an α-amino-3-hydroxy-5-methyl-4-isoxazolepropionic acid (APMA) antagonist [16]. The patients demonstrated improvement in myoclonus with this medication. However, a pragmatic framework for diagnosis and management with a special focus on psychological and social support is direly needed. Genetic testing should always be offered to family members of the affected individuals. Lafora disease is a stealth killer that frequently leads to a premature death. Therefore, it is exceedingly important to glean more information from animal models to make therapeutic advancements based on the pathogenetic aspects of this disease.

## Conclusions

Lafora disease is characterized by a progressive myoclonic epilepsy with onset typically in the second decade of life and a uniformly fatal outcome. This report highlights a remarkably rare presentation of this disorder with hepatic insufficiency, in the absence of characteristic neurological signs and symptoms. It underscores the importance of a high index of clinical suspicion for atypical disease presentations. Biopsy and genetic testing should be stressed to confirm the diagnosis early in the course of the disease. The standard treatment guidelines are not available. Regular clinical follow-up is crucial and psychological support should be offered to the patients. Future research should aim to develop pathogenetically oriented treatments for this difficult-to-treat disease.
